# The projected burden of primary total knee and hip replacement for osteoarthritis in Australia to the year 2030

**DOI:** 10.1186/s12891-019-2411-9

**Published:** 2019-02-23

**Authors:** Ilana N. Ackerman, Megan A. Bohensky, Ella Zomer, Mark Tacey, Alexandra Gorelik, Caroline A. Brand, Richard de Steiger

**Affiliations:** 1 Department of Epidemiology and Preventive Medicine, Monash University, 553 St Kilda Road, Melbourne, Victoria 3004 Australia; 2 Department of Medicine (Royal Melbourne Hospital), The University of Melbourne, Grattan Street, Parkville, Victoria 3050 Australia; 3 The Northern Hospital, 185 Cooper Street, Epping, Victoria 3076 Australia; 4 School of Population and Global Health, The University of Melbourne, 235 Bouverie Street, Carlton, Victoria 3053 Australia; 5 School of Psychology, Australian Catholic University, 215 Spring Street, Melbourne, Victoria 3000 Australia; 6 Department of Surgery, Epworth HealthCare, The University of Melbourne, 89 Bridge Road, Richmond, Victoria 3121 Australia; 7 Australian Orthopaedic Association National Joint Replacement Registry, South Australian Health and Medical Research Institute, North Terrace, Adelaide, South Australia 5000 Australia

**Keywords:** Economic burden of disease, Obesity, Osteoarthritis, Total knee replacement, Total hip replacement

## Abstract

**Background:**

Comprehensive national joint replacement registries with well-validated data offer unique opportunities for examining the potential future burden of hip and knee osteoarthritis (OA) at a population level. This study aimed to forecast the burden of primary total knee (TKR) and hip replacements (THR) performed for OA in Australia to the year 2030, and to model the impact of contrasting obesity scenarios on TKR burden.

**Methods:**

De-identified TKR and THR data for 2003–2013 were obtained from the Australian Orthopaedic Association National Joint Replacement Registry. Population projections and obesity trends were obtained from the Australian Bureau of Statistics, with public and private hospital costs sourced from the National Hospital Cost Data Collection. Procedure rates were projected according to two scenarios: (1) constant rate of surgery from 2013 onwards; and (2) continued growth in surgery rates based on 2003–2013 growth. Sensitivity analyses were used to estimate future TKR burden if: (1) obesity rates continued to increase linearly; or (2) 1–5% of the overweight or obese population attained a normal body mass index.

**Results:**

Based on recent growth, the incidence of TKR and THR for OA is estimated to rise by 276% and 208%, respectively, by 2030. The total cost to the healthcare system would be $AUD5.32 billion, of which $AUD3.54 billion relates to the private sector. Projected growth in obesity rates would result in 24,707 additional TKRs totalling $AUD521 million. A population-level reduction in obesity could result in up to 8062 fewer procedures and cost savings of up to $AUD170 million.

**Conclusions:**

If surgery trends for OA continue, Australia faces an unsustainable joint replacement burden by 2030, with significant healthcare budget and health workforce implications. Strategies to reduce national obesity could produce important TKR savings.

**Electronic supplementary material:**

The online version of this article (10.1186/s12891-019-2411-9) contains supplementary material, which is available to authorized users.

## Background

Increasing levels of obesity, population ageing, and growth in sports-related injuries are all anticipated to manifest in a greater future burden of osteoarthritis (OA) [1–3]. For severe knee or hip OA, total knee (TKR) and hip replacement surgery (THR) have been shown to improve function and quality of life and be cost-effective [4, 5]. The number of TKR and THR procedures has grown steadily over the last two decades in Australia and other developed countries [6–9]. We recently reported a 105% increase in primary TKR utilisation over a 10-year period (2003–2013) in Australia and a 73% increase in THR surgery for OA over this time [10, 11].

Demand for joint replacement surgery is expected to increase in many countries. In the United States (US), Kurtz et al. have predicted growth of 673% for TKR and 174% for THR from 2005 to 2030 [12]. More recently, Inacio et al. have forecast growth in TKR volume in the US of 143 and 855% from 2012 to 2050 using conservative and exponential growth scenarios, respectively [13]. Studies from the UK, Canada, Sweden, New Zealand and Denmark have also predicted increases in joint replacement surgery over the next two decades, although the estimates vary widely [14–18]. In the US, demand for primary TKR and THR among younger age groups has been predicted to grow markedly, with over half of joint replacement recipients expected to be aged under 65 years by the year 2030 [19]. Although TKR and THR projections have been recently published for Australia, neither age-specific nor sex-specific projections were reported [20]. Rising obesity levels are a major driver of TKR rates, as demonstrated by national longitudinal data from the US [21], and warrant particular consideration given the potential impacts on surgery utilisation. A recent large-scale study (*N* = 105,189) also showed that obese individuals in Spain had an at least 2-fold increase (depending on obesity category) in the likelihood of TKR, compared to those of normal weight [22]. Linked Norwegian Arthroplasty Register data (*N* = 225,908) also support a link between weight gain and increased risk of TKR [23]. Reducing the prevalence of obesity at the population level could have important benefits for healthcare systems with regard to fewer TKR procedures [24], although this has not yet been evaluated.

An increasing burden of joint replacement surgery has significant cost and health workforce implications. The cost of a TKR or THR procedure in Australia is estimated at $AUD19,000 to $AUD30,000 per patient [25], with over $AUD1.2 billion spent annually in Australia on OA-related hospital admissions [26]. There are even greater economic implications relating to surgery for younger patients, given the higher risk of multiple revisions [27]. From a health workforce perspective, over one-third of active orthopaedic surgeons in Australia are aged 55 or older and likely to retire within the next 10–15 years [28]. This would undoubtedly impact Australia’s capacity for future provision of joint replacement surgery. An improved understanding of the future national burden of joint replacement in both the public and private health systems is required to ensure that demand can be met and high quality standards can continue. Australia has maintained a validated national joint replacement registry since 2002 (with over 98% coverage of all THR and TKR surgeries performed in public and private hospitals) [9], and these population-level data offer a unique opportunity to generate well-informed projections of national burden.

This study aimed to forecast the number of primary TKR and THR surgeries likely to be performed for OA in Australia to the year 2030 (including age- and sex-specific estimates), and associated costs. It also aimed to model the impact of two contrasting obesity scenarios on future TKR burden.

## Methods

### Design

Epidemiological modelling using available population-level data.

### Data sources

De-identified individual data on patient age, sex, primary diagnosis, procedure year, procedure type (primary TKR or primary THR), procedure side, hospital setting (metropolitan or regional/rural) and hospital type (public or private) were obtained from the Australian Orthopaedic Association National Joint Replacement Registry (AOANJRR). The AOANJRR is a government-funded, national clinical quality registry of all joint replacement procedures performed in Australia. It collects a defined minimum data set that enables short and long-term surgical outcomes to be monitored. Data are collected from all public and private hospitals performing joint replacements, with full national implementation completed in 2002 [29]. Complete national data from all hospitals are available from 2003. All patients with a primary TKR or THR and a diagnosis of OA as recorded in the AOANJRR from 1 January 2003 to 31 December 2013 were included in this analysis.

National population projections, stratified by age and gender, were obtained from the Australian Bureau of Statistics (ABS) [30]. These population projections are based on national Census data and a series of assumptions on future fertility, life expectancy and migration. The moderate projections (series B) were used for this study. Data from the ABS Australian Health Surveys (2007–2008 and 2011–2012) were used to determine the proportion of adults classified as overweight (body mass index (BMI) 25–29.9 kg/m^2^) and obese (BMI ≥ 30 kg/m^2^) and to estimate obesity trends into the future [31, 32].

Procedure costs were obtained from the 2008–2009 National Hospital Cost Data Collection for public and private hospitals [33], using procedure codes for unilateral knee replacement (104Z), unilateral hip replacement (I03B/I03C) and bilateral lower limb joint replacement (101Z). Procedure costs were adjusted for inflation to 2016 Australian dollars using the Total Health Price Index [34].

### Data analysis

Data were categorised into pre-specified age groups for analysis: < 40 years (younger patients), 40–69 years (middle-aged patients), and ≥ 70 years (older patients). Projections of procedure numbers (overall and per 100,000 population) to the year 2030 were based on two different scenarios:

1. age-specific and sex-specific TKR and THR procedure rates in 2013 were projected to continue at a constant rate (‘Scenario 1’); and
2. age-specific and sex-specific TKR and THR procedure rates were projected to continue to increase as they have over the last decade, using Poisson regression analysis (‘Scenario 2’).

Age group, sex and procedure year were included as model covariates. As procedure month data were unavailable, it was not possible to determine if bilateral joint replacement procedures were staged (performed sequentially) or performed simultaneously (on the same date). Adopting a conservative approach (as sequential hospital admissions are more expensive), if two joint replacement procedures were reported for the same patient in the same year within the same hospital setting (public or private), these were assumed to be simultaneous bilateral procedures. All costs were estimated from the perspective of the Australian health system (1 AUD is equivalent to 0.78 USD).

### Sensitivity analyses

Given the known relationship between obesity and increased risk of TKR [35], we also performed sensitivity analyses to evaluate the impact of two contrasting assumptions regarding future population obesity rates. For these analyses, data on obesity trends were obtained from the ABS Australian Health Surveys and the relative risk (RR) of TKR associated with overweight and obesity was obtained from a meta-analysis [35]. We chose pooled risk estimates as these involved a large number of studies and patients, and were similar to the Australian estimates [35]. The population attributable fraction (PAF) for obesity was calculated using a modified Peto-Lopez formula [36], which combines the population distribution of a risk factor and RR of having a single outcome. The PAF formula is shown below:

$$ \boldsymbol{PAF}=\frac{{\boldsymbol{P}}_{\mathbf{0}}\ast \boldsymbol{RR}-{\boldsymbol{P}}_{\mathbf{1}}\ast \boldsymbol{RR}}{{\boldsymbol{P}}_{\mathbf{0}}\ast \boldsymbol{RR}} $$

where ***P***_**0**_ represents the current prevalence of obesity, ***P***_**1**_ represents the projected prevalence of obesity and **RR** is the relative risk of obese and overweight people having TKR.

The total number of TKR cases in 2030 attributable to overweight and obesity was estimated by multiplying the PAF estimates by the projected number of TKR procedures.

In the first sensitivity analysis, we determined the expected number of TKR procedures if obesity rates continued to increase in a linear fashion at the rate witnessed from 2007-2008 to 2011–2012. In the second sensitivity analysis, we determined the number of TKR procedures that could potentially be prevented in 2030 if a proportion (ranging from 1 to 5%) of the Australian population that are currently overweight or obese were to attain a normal BMI.

## Results

### Demographics of primary total hip and knee replacement surgery

Over the 10-year study period there were 350,994 TKR procedures performed on 279,453 people with knee OA in Australia, and 220,916 THR procedures performed on 190,724 people with hip OA. In 2013, the majority of procedures were performed on females, in the private sector, and in metropolitan hospitals (Table 1). From 2003 to 2013 there was a significant decrease in the proportion of patients aged 70 years or older who received TKR or THR (TKR: 53.4% vs 44.5%, *p* < 0.001; THR: 51.2% vs 44.9%, *p* < 0.001). A significant increase in the proportion of procedures performed in private hospitals was also evident over this period (TKR: 65.7% in 2003, increasing to 70.4% in 2013, *p* < 0.001; THR: 65.0% in 2003, increasing to 70.6% in 2013, *p* < 0.001).

**Table 1:** Joint replacement demographics and estimated costs in 2003 and 2013

	Total knee replacement		Total hip replacement	
	2003	2013	2003	2013
Procedures				
Total, *n*	20,986	42,920	15,031	25,945
Females, *n* (%)	12,004 (57.2)	24,305 (56.6)	8192 (54.5)	13,830 (53.3)
Aged ≥70 years, *n* (%)	11,206 (53.4)	19,115 (44.5)	7701 (51.2)	11,655 (44.9)
Private hospital, *n* (%)	13,650 (65.0)	29,783 (69.4)	9581 (64.1)	18,163 (70.0)
Metropolitan hospital, *n* (%)	13,687 (65.2)	27,557 (64.6)	10,107 (67.2)	17,556 (67.7)
> 1 procedure in same year^a^, *n* (%)	2824 (13.5)	6978 (16.3)	860 (5.7)	1688 (6.5)
Estimated costs^b^				
Overall health system	$448 million	$905 million	$364 million	$625 million
Private health system only	$272 million	$591 million	$227 million	$429 million
Public health system only	$176 million	$314 million	$137 million	$196 million

^a^Two or more procedures in the same year and same hospital setting were classified as bilateral simultaneous procedures for costing purposes

^b^All costs are reported in 2016 Australian dollars

### Procedure rates and costs in 2003 and 2013

**Fig. 1:**
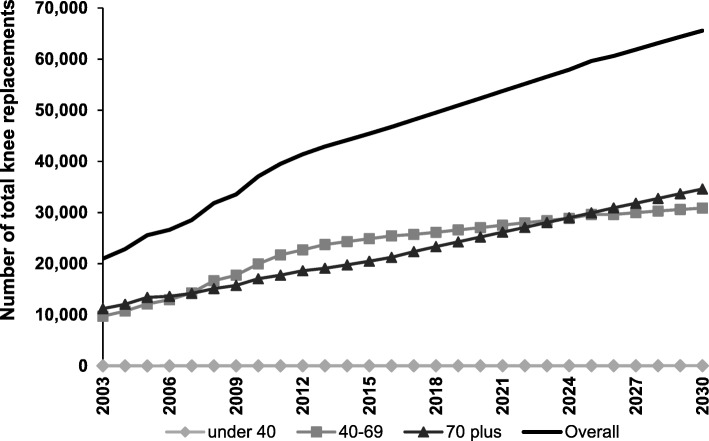
Growth in number of total knee replacements from 2003 to 2030 under Scenario 1. Number of total knee replacement procedures for 2003–2013 is based on numbers reported to the AOANJRR. Number of procedures from 2014 onwards is based on projections under Scenario 1

In 2003, the rate of TKR in Australia was 123 per 100,000 population. By 2013, the rate of TKR had increased to 213 per 100,000 population, with growth in the number of procedures for the 40–69 and ≥ 70 age groups and overall (Fig. 1). The largest absolute growth in TKR procedures was evident for people aged 40–69 years (increase of 14,014 procedures), while there was little change in the number of procedures for people aged under 40 years (increase of 11 procedures). Sex-specific graphs are provided in Additional file 1. In 2003 the total estimated cost of TKR was $AUD448 million, rising to $AUD905 million in 2013.

**Fig. 2:**
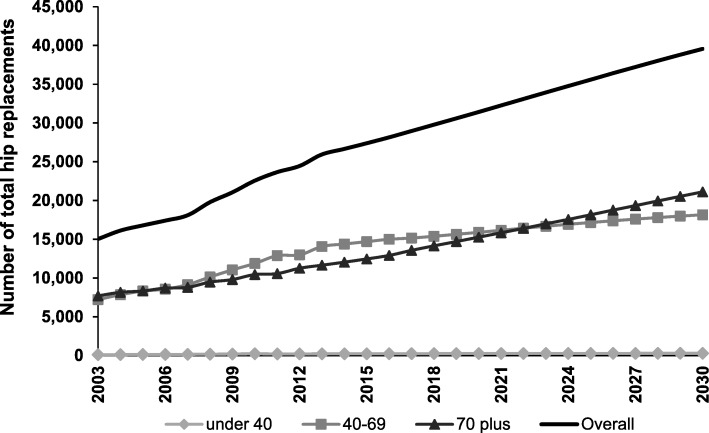
Growth in number of total hip replacements from 2003 to 2030 under Scenario 1. Number of total hip replacement procedures for 2003–2013 is based on numbers reported to the AOANJRR. Number of procedures from 2014 onwards is based on projections under Scenario 1

From 2003 to 2013, the rate of THR in Australia increased from 88 to 129 per 100,000 population. As shown in Fig. 2, the number of procedures grew substantially for those aged 40–69 years (increase of 6848 procedures) and those aged ≥70 years (increase of 3954 procedures). There was little change for the < 40 age group (increase of 112 procedures). Sex-specific analyses showed that males and females aged 40–69 years demonstrated similar absolute increases in the number of THR procedures from 2003 to 2013 (Additional file 2). From 2003 to 2013, the total estimated cost of THR increased from $AUD364 million to $AUD625 million per year.

### Projected procedure rates and costs to 2030

Assuming rates of procedures in 2013 remained constant over time (Scenario 1), the incidence of TKR is predicted to be 65,569 procedures by 2030, or 248 TKRs per 100,000 population, at an estimated cost of $AUD1.38 billion to the healthcare system. Under this assumption, the incidence of THR procedures is predicted to reach 39,567 procedures by 2030, or 150 THRs per 100,000 population, at an estimated cost of $AUD953 million. Of the total forecast costs for THR and TKR in the year 2030, 66.7% of these costs (equating to $AUD1.56 billion) relate to the private hospital sector.

As Scenario 1 was driven by projected population growth, the greatest increase in procedures from 2013 to 2030 was among people aged 70 years and older. For TKR, the ≥70 year age group accounted for an additional 15,505 procedures (Fig. 1). For THR, this age group accounted for an additional 9461 procedures (Fig. 2).

**Fig. 3:**
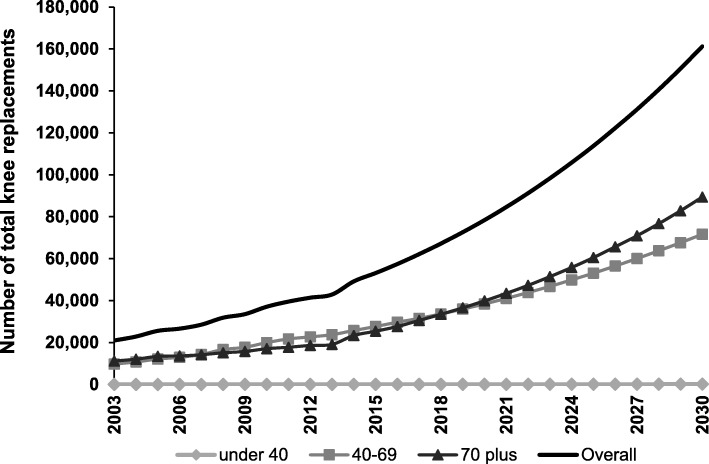
Growth in number of total knee replacements from 2003 to 2030 under Scenario 2. Number of total knee replacement procedures for 2003–2013 is based on numbers reported to the AOANJRR. Number of procedures from 2014 onwards is based on projections under Scenario 2

**Fig. 4:**
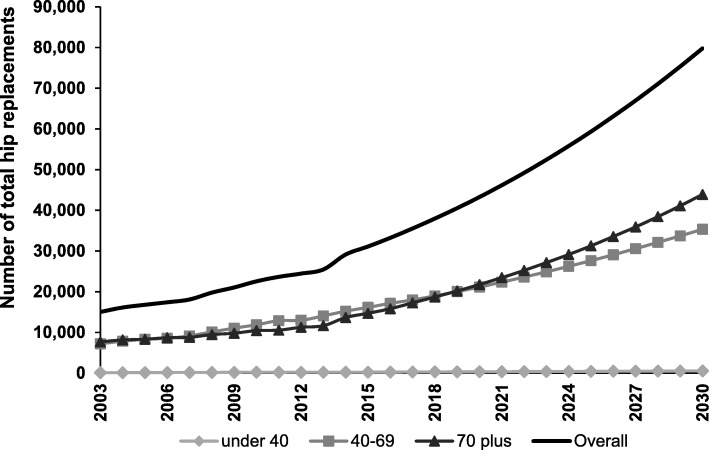
Growth in number of total hip replacements from 2003 to 2030 under Scenario 2. Number of total hip replacement procedures for 2003–2013 is based on numbers reported to the AOANJRR. Number of procedures from 2014 onwards is based on projections under Scenario 2

For Scenario 2 (which modelled increasing rates of surgery over time), the incidence of TKR was estimated to reach 161,231 procedures (Fig. 3), or 609 procedures per 100,000 population by 2030. This represents a 276% increase from 2013 procedure numbers. The incidence of THR was estimated to increase to 79,795 procedures by 2030 (Fig. 4), or 302 procedures per 100,000 population. This represents a 208% increase from 2013 procedure numbers. Sex-specific projections for TKR are provided in Additional file 3 and sex-specific projections for THR are provided in Additional file 4. Under Scenario 2, the cost to the healthcare system is forecast to be $AUD3.40 billion for TKR and $AUD1.92 billion for THR. It is estimated that $3.54 billion of these combined costs would be borne by the private hospital sector.

### Sensitivity analyses

In 2007–2008, the prevalence of overweight and obesity in Australia was 67.7% for men and 54.6% for women, according to national measured height and weight data [31]. In 2011–2012, this increased to 70.3% for men and 56.2% for women [37]. Assuming linear growth in overweight and obesity rates over time (which equates to an annual increase of 0.65% for men and 0.40% for women), 82.7% of Australian men and 63.8% of Australian women will be overweight or obese by the year 2030. Under Scenario 1 (assuming a constant rate of TKR), this would lead to an estimated incidence of 286 TKR procedures per 100,000 population by 2030, or an extra 10,033 procedures, resulting in additional costs to the health system of $AUD212 million (Table 2). Under Scenario 2 (assuming an increasing rate of TKR), the projected increase in overweight and obesity rates would result in 24,707 extra procedures in 2030 at an additional cost of $AUD521 million.

**Table 2:** Total knee replacement projections and costs in 2030 with changes in obesity rates

	Projected burden		Change in burden based on changes in obesity rates	
Projection scenario	Procedures (*n*)	Cost	Procedures (*n*)	Cost
Scenario 1 (constant TKR rate)				
Base projection	65,569	$1.38 billion		
Sensitivity analysis - increasing overweight/obesity	75,602	$1.59 billion	+ 10,033	+$212 million
Sensitivity analysis - decreasing overweight/obesity				
1%	64,913	$1.37 billion	−656	-$14 million
2%	64,257	$1.35 billion	− 1312	-$28 million
3%	63,602	$1.34 billion	− 1967	-$41 million
4%	62,946	$1.33 billion	− 2623	-$55 million
5%	62,290	$1.31 billion	− 3279	-$69 million
Scenario 2 (increasing TKR rate)				
Base projection	161,231	$3.40 billion		
Sensitivity analysis - increasing overweight/obesity	185,938	$3.92 billion	+ 24,707	+$521 million
Sensitivity analysis - decreasing overweight/obesity				
1%	159,619	$3.37 billion	−1612	-$34 million
2%	158,006	$3.33 billion	− 3225	-$68 million
3%	156,394	$3.30 billion	− 4837	-$102 million
4%	154,782	$3.26 billion	− 6449	-$136 million
5%	153,169	$3.23 billion	−8062	-$170 million

All costs are reported in 2016 Australian dollars

Assuming the rate of overweight or obesity in 2011–2012 decreases between 1 and 5% in 2030, the incidence of TKR would be between 235 and 245 procedures per 100,000 population under Scenario 1, or between 656 and 3278 fewer procedures. This would result in total cost savings of up to $AUD69 million (Table 2). Under Scenario 2, the same reduction in overweight and obesity would result in a TKR incidence of between 579 and 603 procedures per 100,000 population, or between 1612 and 8062 fewer procedures. This would equate to total cost savings of up to $AUD170 million.

## Discussion

This study has produced comprehensive estimates of the future burden of TKR and THR for OA in Australia (by age, sex and overall, as well as by healthcare sector), using a well-validated national dataset that includes all joint replacement procedures performed in this country. According to our projections, Australia faces a potentially unsustainable joint replacement burden by 2030 which requires significant investment in public and private health systems and health workforce training. Based on growth in surgery rates over a decade, TKR procedures for OA are expected to increase by 276% (from 42,920 procedures in 2013 to 161,231 in 2030) while THR procedures for OA are predicted to rise by 208% (from 25,945 procedures in 2013 to 79,795 in 2030). The total cost to the health system is forecast to exceed $5.32 billion in 2030. These estimates can be used to facilitate healthcare resource planning and inform health policymakers and public health practitioners about future national demand for joint replacement.

Much of the predicted growth in TKR and THR is driven by population ageing. From 2003 to 2013, the Australian population aged 40 years and over increased from 8.7 million (representing 51% of the total population) to 10.7 million (representing 53.1% of the total population). By 2030, this age group is expected to number 14.8 million, or 55.8% of the total population. Meeting the large growth in surgical demand will prove challenging for Australia, given the ageing surgical workforce [28]. Although joint replacement should be reserved for severe, end-stage OA, if individuals choose to undergo joint replacement for milder symptoms [38] this could further augment demand for surgery. We observed an initial sharp increase in TKR procedures from 2003 which appeared to level off by 2013, particularly for the 40–69 age group. This trend was also identified in our state-level analyses [39], and may relate to ‘catch up’ of previous unmet need for surgery following the introduction of financial incentives designed to improve the uptake of private health insurance. Our projections do not take into account the potential impact of rising rates of lower limb sports-related injuries [3], and this has specific relevance for TKR as injury has been identified as a major contributor to knee OA [40]. Further epidemiological data are needed to quantify the increased risk of TKR and THR associated with sports injury, and this research is currently underway.

Although a range of international studies has reported projections of joint replacement burden, comparisons are difficult given differing samples, methodological approaches and timeframes for analysis. Our methods differ substantially to those used for a recent study that projected growth in TKR and THR rates in Australia from 2014 to 2046 [20]. We limited our data inputs to procedures performed for OA (11% of THR procedures in Australia are performed for non-OA diagnoses [41] including fractured neck of femur) and we calculated age- and sex-specific rates to examine subgroup trends in joint replacement growth. Despite methodological differences, our 2030 Scenario 2 projections for TKR and THR fall clearly within the 95% prediction intervals reported by Inacio et al. for that year [20]. Variability in projections between countries could relate to differences in obesity rates, data accuracy, and health system differences, particularly for countries with mixed public-private systems. The study by Kurtz et al. [12] used discharge records from the US Nationwide Inpatient Sample, representing about 20% of all community hospitals. Based on surgery trends from 1990 to 2003, the researchers predicted a 673% increase in TKR and a 174% increase in THR from 2005 to 2030. The potential impact of changing obesity rates was not examined. Using a general practice database covering approximately 10% of the total population, Culliford et al. estimated a 26% increase in TKR and also for THR in the UK from 2015 to 2030 (assuming that 2010 surgery rates remained constant) [17]. Using similar methods (assuming the 2013 surgery rate remained constant), we projected a 53% growth for TKR and also for THR in Australia, based on population growth. The differences in our static-rate projections may partly relate to differences in projected growth (and population structure) for the Australian and UK populations over time [30, 42]. The UK study also predicted that TKR would increase by a further 7% if BMI proportions continue to increase over time. Neither the US nor UK studies was limited to patients with a primary diagnosis of OA. Denmark and Sweden have also predicted increased demand for THR by 2020 and 2030, respectively [14, 16], while projections of TKR and THR burden in New Zealand to 2026 have also been published [15]. While this paper focuses on the cost burden associated with joint replacement surgery, the benefits from surgery are substantial, as highlighted by numerous studies assessing the cost-effectiveness of THR and TKR procedures [4, 43, 44]. Most recently, Elmallah et al. showed that THR and TKR were associated with lifetime quality-adjusted life year (QALY) gains of 2.07 and 1.85, respectively [44]. Cost-effectiveness was demonstrated by an incremental cost-effectiveness ratio of approximately $US39,000 per QALY for THR and approximately $US43,000 per QALY for TKR [44], which falls well below the arbitrary cost-effectiveness threshold of $US50,000 to $US150,000 [45]. Using a discrete-event simulation model, Higashi et al. estimated that population health gains from joint replacement in Australia were equivalent to 115,000 disability-adjusted life years (DALYs) averted for THR and 113,000 DALYs averted for TKR [46]. These figures highlight the value of joint replacement procedures at the population level.

Quantifying the potential impact of reducing obesity on the projected healthcare costs of TKR provides a strong policy and public health argument for supporting population-level weight loss campaigns and individual-level interventions. ABS data show that the proportion of Australians who are overweight or obese increased from 61.2% in 2007–2008 to 62.8% in 2011–2012 [37]. We estimated this would exceed 70% by 2030, resulting in a further 15% growth in TKR procedures. TKR surgery for people who are obese can produce substantial improvements in pain and function (comparable in magnitude to improvements experienced by people in the normal weight range [47]) and may indeed be clinically warranted. However, there are increased surgery and episode of care costs for obese patients undergoing this procedure [48]. It is also possible that as obesity rates increase over time, TKR costs may accelerate faster than monetary inflation, with significant health budget implications. It is unknown whether a reduction of overweight and obesity by 5% at the population level is achievable, so we modelled the benefits on a sliding scale at varying degrees of weight loss. However, a US randomised controlled trial involving 454 overweight and obese older participants with radiographic knee OA found that the combined diet/exercise intervention group had a mean weight loss of 11.4% of body mass, and the diet-only intervention group reported a mean weight loss of 9.5% of body mass over the 18-month study [49]. If such dramatic weight loss results could be achieved in ‘real-world’ settings, then a large proportion of overweight people could be transitioned into a normal BMI category. In the present study we did not explore the potential impact of changes in obesity levels on THR, as no consistent association between BMI and risk of hip OA or THR has been demonstrated [50–52].

A key strength of this study was our use of national registry data collected over a 10-year period to project the future incidence of TKR and THR for OA. Given external data validation processes, we are confident the AOANJRR dataset provides a comprehensive picture of joint replacement utilisation in Australia. We also used two different scenarios for projecting future burden, which helps to quantify potential uncertainty in our estimates. However, we acknowledge several limitations to our methods. As patient-level data on BMI were not available, we assumed that overweight and obesity rates matched Australian population rates. As people undergoing TKR may have higher rates of overweight and obesity [53], this is a conservative assumption. We also assumed linear growth in obesity rates over time, consistent with previous methods [54]. Administrative data (average costs per TKR or THR admission) were used to impute costs for the index procedure and we did not have access to patient-level costing data relating to post-operative complications or revision surgery. We also conservatively assumed that bilateral procedures were performed simultaneously and recognise that staged procedures are more costly. However, as relatively few bilateral procedures are performed (representing 16.3% of all TKRs and 6.5% of all THRs in 2013) we do not consider this to be problematic. Our study focused on direct costs to the health system and did not include personal (eg out-of-pocket healthcare costs) and societal costs (eg lost productivity and carer time), which are likely to be substantial. Finally, it is possible that the development of new medical interventions for OA could allay some of the future burden of joint replacement although this would be unlikely over the study forecast period, and that initiatives to reduce unit cost per joint replacement episode (for example, lower implant costs, use of day surgery, and home-based rehabilitation) could also reduce the future economic burden but were not the focus of this research.

## Conclusions

In conclusion, the burden of TKR and THR in Australia (in terms of number of procedures and healthcare costs) is forecast to increase substantially by 2030. Based on recent growth in surgery rates, the annual incidence of TKR and THR is predicted to exceed 161,000 and 79,000 procedures, respectively, by the year 2030. The projected cost to the health system will total $5.32 billion by 2030, of which $3.54 billion relates to the private healthcare sector. Increasing rates of obesity could result in nearly 25,000 additional TKRs annually by 2030, while strategies to reduce national obesity could produce important TKR savings. These projections can be used to inform future healthcare resource planning, including health workforce development, to ensure capacity to meet joint replacement demand.

## Additional files

Additional file 1: **Figure A1:** Description of data: Fig. A1. Growth in number of total knee replacements from 2003 to 2030 under Scenario 1, by sex (DOCX 26 kb)

Additional file 2: **Figure A2**. Description of data: Fig. A2. Growth in number of total hip replacements from 2003 to 2030 under Scenario 1, by sex (DOCX 26 kb)

Additional file 3: **Figure A3**. Description of data: Fig. A3. Growth in number of total knee replacements from 2003 to 2030 under Scenario 2, by sex (DOCX 26 kb)

Additional file 4: **Figure A4**. Description of data: Fig. A4. Growth in number of total hip replacements from 2003 to 2030 under Scenario 2, by sex (DOCX 26 kb)

## Abbreviations

ABS: Australian Bureau of Statistics
AOANJRR: Australian Orthopaedic Association National Joint Replacement Registry
AUD: Australian dollar
BMI: Body mass index
OA: Osteoarthritis
PAF: Population attributable fraction
RR: Relative risk
THR: Total hip replacement
TKR: Total knee replacement
