# Supplementary material for: The projected burden of primary total knee and hip replacement for osteoarthritis in Australia to the year 2030
Source: BMC Musculoskelet Disord. 2019 Feb 23;20:90. doi: 10.1186/s12891-019-2411-9 (PMC6387488; doi:10.1186/s12891-019-2411-9)
Supplement: Supplementary file 2 — Figure A2. Description of data: Fig. A2. Growth in number of total hip replacements from 2003 to 2030 under Scenario 1, by sex (DOCX 26 kb) [file 12891_2019_2411_MOESM2_ESM.docx]

**Additional file 2**

**The projected burden of primary total knee and hip replacement for osteoarthritis in Australia to the year 2030**

Ilana N Ackerman, Megan A Bohensky, Ella Zomer, Mark Tacey, Alexandra Gorelik,

Caroline A Brand and Richard de Steiger

**Figure A2. Growth in number of total hip replacements from 2003-2030 under Scenario 1, by sex**

Number of total hip replacement procedures for 2003-2013 is based on numbers reported to the AOANJRR

Number of procedures from 2014 onwards is based on projections under Scenario 1
